# The impact of the military mission in Afghanistan on mental health in the Canadian Armed Forces: a summary of research findings

**DOI:** 10.3402/ejpt.v5.23822

**Published:** 2014-08-14

**Authors:** Mark A. Zamorski, David Boulos

**Affiliations:** Canadian Forces Health Services Group, Ottawa, ON, Canada

**Keywords:** Military personnel, Canada, stress disorders, post-traumatic, combat, epidemiology

## Abstract

**Background:**

As Canada's mission in Afghanistan winds down, the Canadian Forces (CF) are reflecting on the psychological impact of the mission on more than 40,000 deployed personnel.

**Methods:**

All major CF studies of mental health outcomes done before and during the Afghanistan era are summarized, with an eye toward getting the most complete picture of the mental health impact of the mission. Studies on traumatic brain injury (TBI), high-risk drinking, and suicidality are included given their conceptual link to mental health.

**Results:**

CF studies on the mental health impact of pre-Afghanistan deployments are few, and they have inadequate detail on deployment experiences. Afghanistan era findings confirm service-related mental health problems (MHPs) in an important minority. The findings of the studies cohere, both as a group and in the context of data from our Allies. Combat exposure is the most important driver of deployment-related MHPs, but meaningful rates will be found in those in low-threat areas. Reserve service and cumulative effects of multiple deployments are not major risk factors in the CF. Many deployed personnel will seek care, but further efforts to decrease the delay are needed. Only a fraction of the overall burden of mental illness is likely deployment attributable. Deployment-related mental disorders do not translate into an overall increase in in-service suicidal behavior in the CF, but there is concerning evidence of increased suicide risk after release. TBI occurred in a distinct minority on this deployment, but severe forms were rare. Most TBI cases do not have persistent “post-concussive” symptoms; such symptoms are closely associated with MHPs.

**Conclusion:**

The mental health impact of the mission in Afghanistan is commensurate with its difficult nature. While ongoing and planned studies will provide additional detail on its impacts, greater research attention is needed on preventive and therapeutic interventions.

Since late 2001, more than 40,000 Canadian military personnel have deployed in support of its mission in Afghanistan. As the current phase of the Afghanistan mission winds down, Canada is reflecting on the accomplishments and costs of the mission.

This paper summarizes research on one of these costs: The mission's impact on the mental health of those deployed. An overview of the Canadian Forces (CF) and its missions over the past decades will be provided. Next, a summary of key studies done prior to the CF's involvement in Afghanistan is presented. Studies on the impact of the Afghanistan mission are then reviewed. Studies are reviewed by describing the study population, the means and context of assessment, prevalence rates, correlates of mental health problems (MHPs), key study limitations, and the key findings and implications of each study. This paper concludes with a brief synthesis of the thrust of the findings of the major studies.

The focus will be on mental disorders as opposed to dimensional outcomes like well-being. As the most common service-related mental disorders in the CF, PTSD and depression will be the key mental health outcomes (Boulos & Zamorski, 2013). Findings on high-risk drinking, suicidality, and traumatic brain injury (TBI) (Bryant, 2011) will be discussed given their linkage to mental health. Relatively little of this work has been published in the peer-reviewed literature; unpublished studies are available on request.

Our overall approach reflects our perspective that understanding the mental health impacts of deployment hinges upon a deep understanding of the full range of factors contributing to the apparent prevalence of MHPs (e.g., the baseline prevalence of mental disorders in those who deployed, their past deployment experiences, their experiences on the deployment in question, the strength of the overall mental health system, the context of the assessment of mental health, and the specific assessment methods used). To draw attention to the importance of these factors in driving the results, the studies are presented one-by-one as opposed to thematically, in the hope that this will facilitate comparison with studies done in other nations.

## The CF context

### Composition

Currently, the CF consists of approximately 68,000 Regular Force (RegF) personnel and 27,000 Primary Reserve Force (ResF) personnel. Most deployers are RegF personnel, so the following results focus on them. In the RegF, personnel are recruited with the intent of a full military career if desired. As a result, only about 10% are under age 25. The RegF is about 15% female.

### Pre-Afghanistan operational history

**Fig. 1:**
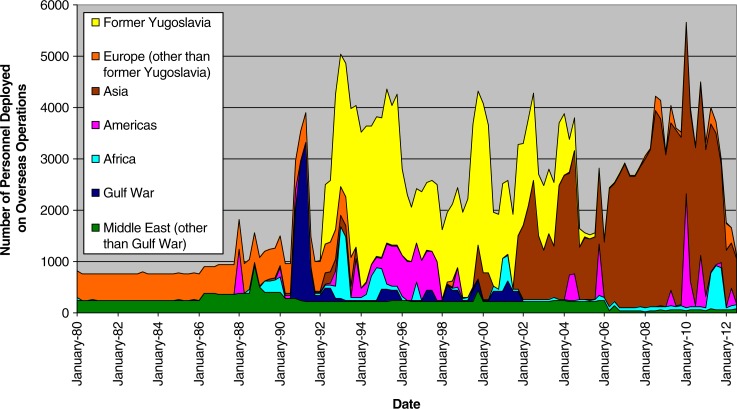
Number of Canadian Forces personnel deployed on international operations, by geographical area, 1980–2012.

Figure 1 summarizes the operational history of the CF from 1980 to 2012 by geographic location. Prior to the Afghanistan mission (2001–2014), Canada was largely involved in United Nations peacekeeping and humanitarian missions, mainly in the former Yugoslavia, Cyprus, the Mideast, Africa, and Haiti. These operations varied dramatically with respect to exposure to potentially traumatic events.

### Afghanistan operational history

**Fig. 2:**
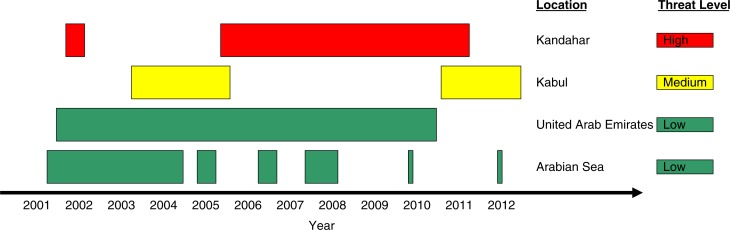
Temporality of major deployments in support of the mission in Afghanistan, by geographical area, 2001–2012.

CF deployments are typically 6 months in duration, with about 24 months of dwell time in-garrison between deployments. Figure 2 summarizes the timing and location of major CF deployments in the support of the mission. The threat level varied by deployment location, with most of those in the Arabian Gulf and the United Arab Emirates having a low threat level, those in Kandahar having a high threat level, and those in Kabul having an intermediate threat level. Deployments to Kandahar represent the majority of all deployments in support of the mission, and nearly all of the 158 CF deaths occurred there.

### The CF's mental health system

RegF members access mental health care primarily through primary care providers and mental health professionals at bases across Canada; specialty mental health services require referral, but social workers, mental health nurses, and addictions counselors staff open-access clinics. ResF members receive their care through the civilian health care system, though occupational health care and treatment of illness and injuries resulting from military service is provided by the CF.

In response to deficiencies identified in the late 1990s, the CF's mental health system has evolved dramatically over the past decade. The clinical mental health system was reinforced by standing-up regional Operational Trauma and Stress Support Centres for the evaluation and treatment of operational MHPs, by introducing a team-based, interdisciplinary assessment and treatment model, and by doubling the number of CF mental health personnel. Barriers to care were addressed through expansion of mental health screening, strengthening of confidentiality and career protections for those seeking mental health care, and de-stigmatization efforts. Finally, mental health and resilience training across the career-cycle and deployment cycle was institutionalized in the form of the Road to Mental Readiness (R2MR) program (Canadian Armed Forces [CAF], 2013), which targets performance under adversity, enhancement of mental health literacy, and overcoming barriers to care.

## Pre-Afghanistan era findings

Table 1 provides a summary of the population, methods, and key results of the larger studies discussed below. Pre-Afghanistan findings are presented in order to give a sense of the past operational experience and baseline mental health of personnel who ultimately deployed in support of that mission.

**Table 1:** Summary of key survey data sources and findings

Data source (abbreviation)	Population and context	Year(s) of data collection	Method	Sampling approach and sample size (response rate)	Key findings	Comments
Goss Gilroy Health Study of the Canadian Forces Personnel Involved in the 1991 Conflict in the Persian Gulf^1^	Currently-serving personnel and veterans who deployed as part of the 1990–1991 Persian Gulf War, along with matched non-deployed controls	1997	Voluntary, anonymous paper survey	Census of all Gulf War personnel and veterans, *N*=3,100 (73%); stratified random sample of matched non-deployed controls, *N*=3,400 (60%)	Point prevalence of PTSD in Gulf War Veterans (GWVs) (PCL-M^2^≥50)=2.1–2.5%; major depression (PRIME MD^3^)=14.9–18.9%; alcohol abuse (CAGE/PRIME MD^4^)=13.7–14.5%	Significantly higher rate of depression in *GWV*’s; no increased risk of alcohol abuse
Canadian Forces Supplement to the Canadian Community Health Survey Cycle 1.2—Mental Health and Well-being (CCHS 1.2)^5^	In-garrison, regular and reserve forces (RegF, ResF)	2002	Voluntary, confidential computer-assisted personal interview; performed by Statistics Canada on behalf of the CF	Stratified random sample, *N*=8,400 (81%)	12-month prevalence in RegF members using WMH-CIDI^6^ for PTSD=2.8%; major depression=7.6%; alcohol dependence=4.0%; suicidal ideation=4.2%	Combat or exposure to atrocities was associated with increased risk of a number of mental disorders, but these accounted for little of the overall burden of mental illness
2004 Health and Lifestyle Information Survey (HLIS)^7^	In-garrison, RegF only	2004	Voluntary, anonymous paper survey	Stratified random sample, *N*=3,000 (62%)	12-month prevalence of depression (CIDI-SF)^8^=7.1% members; 12-month suicidal ideation=3.2%; current high-risk drinking (AUDIT^9^≥8)=13%	PTSD was not assessed in 2004
2008/2009 Health and Lifestyle Information Survey^10^	In-garrison, RegFs only	2008–2009	Voluntary, anonymous paper survey	Stratified random sample, *N*=2,300 (53%)	Point prevalence of PTSD (PC-PTSD^11^≥3)=8.1%; 3% reported having been diagnosed with PTSD as a chronic condition; 12-month prevalence of depression (CIDI-SF)=7.4%; 12-month suicidal ideation=3.2%; current high-risk drinking (AUDIT^9^≥8)=20%	No change in depression or suicidal ideation since 2004; increase in high-risk drinking since 2004; PTSD not associated with recent deployment
Survey on Transition to Civilian Life^12^	Veterans released from military service from 1998–2007	2010	Computer-aided telephone interview	Stratified random sample, *N*=3,500 (71%)	Self-report of having been diagnosed with a mental health problems (MHPs) as a chronic condition: PTSD=11%; other anxiety disorders=10%; depression or anxiety=20%; 12-month suicidal ideation=6%	
Operational Mental Health Assessment (OMHA)^13^	Just past the mid-point of a 7-month deployment in Kandahar, Afghanistan; approximately 60% of respondents spent most of their time in forward areas	2010	Voluntary, anonymous paper or electronic survey based on the US Army's Soldier Well-being Survey^14^	Near census of a single troop rotation, *N*=1,600 (57%)	Current symptoms of PTSD (PCL-C^15^≥50)=4.6%; depression (PHQ^16^)=4.5%	Combat exposure and home-front stressors were independently associated with MHPs
Enhanced Post-deployment Screening (EPDS)^17^	3 to 6 months after return from 6-month deployments in support of the CF's mission in Kandahar, Afghanistan	2005–2010	Confidential screening questionnaire with validated instruments followed by a mandatory 40-min interview with a mental health professional	Near census, *N*=17,600 (77%)	Prevalence of current symptoms of PTSD (PCL-C^15^≥50)=3.4%; depression (PHQ^16^)=3.5%; high-risk drinking (AUDIT^9^≥8) in 17%	Combat exposure was strongly associated with MHPs; multiple deployments had very small incremental risk of PTSD and/or depression; prevalence of PTSD and/or depression appears to be declining over time
Canadian Forces Base Gagetown Cohort Study^18^	Retrospective cohort study of deployment-related mental disorders for personnel deployed to Kandahar Province	2007–2011	Clinical diagnoses abstracted from electronic health record	Census of personnel who deployed from a single CF base on a single rotation to Kandahar in 2007	Over 4 years of follow-up, 20% were diagnosed with PTSD^19^ by the CF	8% had been medically released, 15% had permanent duty limitations, and 62% had temporary duty limitations
Operational Stress Injury Cumulative Incidence Study^20^	Retrospective cohort study of personnel deployed in support of the mission in Afghanistan	2001–2011	Clinical diagnoses abstracted from medical records	Stratified random sample (*N*=2,000) of personnel deployed anywhere in Southwest Asia from 2001–2008	Over a median period of 4 years of follow-up, cumulative incidence of PTSD^19^=8% and of other Afghanistan-related mental disorders=5.2%	High threat deployment location was a powerful risk factor for deployment-related mental disorders
Self-reported TBI Surveillance Study^21^	3 to 6 months after return from 6-month deployments in support of the CF's mission in Kandahar, Afghanistan	2009*–*2011	Confidential screening questionnaire with validated instruments followed by a mandatory 40-min interview with a mental health professional.	Near census, *N*=10,000 (76%)^22^	*5% screened positive for TBI on the DVBIC* 23 screening questionnaire	Persistent symptoms were seen in a minority but were strongly related to MHPs

1 Goss Gilroy, Inc. (1998).

2 PTSD Checklist, Military Version (Weathers, Litz, Herman, Huska, & Keane, 1993).

3 Primary Care Evaluation of Mental Disorders questionnaire (Spitzer et al., 1994) as modified for the Iowa Persian Gulf Veteran Study (The Iowa Persian Gulf Study Group, 1997).

4 Symptoms of alcohol dependence were assessed using a combination of the CAGE questionnaire (Mayfield, McLeod, & Hall, 1974) and the PRIME MD questionnaire (Spitzer, Kroenke, & Williams, 1999), using the algorithm of the Iowa Persian Gulf Study Group (1997).

5 Documentation of study methods can be found at Statistics Canada (2004). An almost identical study (Statistics Canada, 2002) was done on the Canadian general population at the same time, permitting comparisons between the civilian and military populations.

6 World Mental Health—Composite International Diagnostic Interview (Kessler et al., 2004).

7 DND (2005).

8 Composite International Diagnostic Interview—Short Form (Kessler, Andrews, Mroczek, Ustun, & Wittchen, 1998).

9 Alcohol Use Disorders Identification Test (Bohn, Babor, & Kranzler, 1995).

10 DND (2010).

11 Prins et al. (2003).

12 Thompson et al. (2011).

13 Garber et al. (2012).

14 Office of the Surgeon General (2006).

15 Weathers, Litz, Huska, and Keane (1994).

16 Spitzer et al. (1999).

17 Zamorski (2011a).

18 Sedge, Devlin, and Joshi (2012).

19 DSM-IV diagnostic criteria (American Psychiatric Association [APA], 2000) are used for mental disorders in the CF.

20 Boulos and Zamorski (2013).

21 Zamorski (2009).

22 Self-reported TBI data pertain to a sub-sample of 9,900 of those who completed a version of the screening questionnaire that had TBI screening items.

23 Defense and Veterans Brain Injury Center screening questionnaire (Warden & Ryan, 2005).

### The Health Study of the Canadian Forces Personnel Involved in the 1990–1991 Conflict in the Persian Gulf (1997)

A 1997 survey of CF Gulf War Veterans (GWVs) and non-deployed controls confirmed that GWVs had a higher risk of MHPs, including major depression and PTSD, but not alcohol abuse (Goss Gilroy Inc., 1998). Rates of current symptoms of PTSD were low in absolute terms in those with and without other deployments (2.5 and 2.1%, respectively); current symptoms of major depression were more prevalent (18.9 and 14.9%, respectively). Independent predictors of MHPs tended to include low rank, Army service, and low household income.

This study included released members, giving a more complete picture of the mental health impact of the operation. The matched comparison group permits better judgment of the absolute and relative burden of deployment-related MHPs. However, it suffers from the usual problems of cross-sectional, self-report surveys, such as non-response bias, social desirability bias, difficulty distinguishing between causal and non-causal association, and misclassification related to limitations in the survey instruments used.

The essential conclusions of this study were that PTSD and depression were reasonably prevalent in those with and without Gulf War deployments, that depression was associated with that deployment but PTSD was not, but that the magnitude of the increased risk in depression in those with Gulf War service was small.

### The 2002 Canadian Forces Supplement to the Canadian Community Health Survey Cycle 1.2—Mental Health and Well-being

A 2002 prevalence survey (Statistics Canada, 2004) using a version of the Composite International Diagnostic Interview (Kessler et al., 2004) of approximately 8,400 RegF and ResF personnel showed that RegF personnel suffered from a broad range of common 12-month mental disorders at prevalence rates that were similar to or greater than those of the general population (Zamorski, Uppal, Boddam, & Gendron, 2006a). The 12-month prevalence rate of major depression was 7.6% in the RegF vs. 5.5% in the age-sex adjusted general population (Statistics Canada, 2003); after adjustment for other sociodemographic confounders, the odds of 12-month depression was 2.1 times that of the general population (Zamorski et al., 2006a). A small excess in 12-month panic disorder was also seen. After adjustment, prevalence rates of alcohol dependence, social phobia, and generalized anxiety disorder were similar in RegF personnel and civilians. Twelve-month disorder prevalence rates in the ResF were well below those of the RegF (adjusted odds ratios of ~0.4), with the exception of alcohol dependence (Zamorski, Ng, Uppal, Boddam, & Gendron, 2006b). Twelve-month PTSD prevalence was 2.8 and 1.2% in the RegF and ResF, respectively (Statistics Canada, 2003). PTSD was not assessed in the general population, so prevalence comparisons are not possible.

Risk factors for 12-month mental disorders tended to include sex (greater risk of mood and anxiety disorders in women, greater risk of alcohol dependence in men), youth, being unmarried, and low income (Zamorski et al., 2006a). While less than half of those with a 12-month MHP had sought care for it, CF members were more likely than their civilian counterparts to have done so (Zamorski et al., 2006a). Nevertheless, the median delay to first care for deployment-related PTSD was 5.5 years (Fikretoglu, Liu, Pedlar, & Brunet, 2010).

The number of career deployments had a univariate association with both 12-month and lifetime PTSD, but this association did not persist after adjustment for confounders (Zamorski et al., 2006b). Using a wiser approach that accounted for different *types* of deployment, Sareen et al. (2007) found that *combat* deployments and deployments in which there was *exposure to atrocities* (but not deployment in their absence) *were* associated with an increased risk of any of six 12-month disorders (adjusted odds ratio for combat=1.4 and for exposure to atrocities=1.8). Depression and PTSD (but not panic disorder, social phobia, generalized anxiety disorder, or alcohol dependence) were independently associated with combat; all but alcohol dependence were associated with exposure to atrocities. Twelve-month suicidal ideation was no more common than in civilians, though CF personnel who had witnessed atrocities had a slightly increased risk. Lifetime suicide attempts (but not ideation) were less common in CF personnel (Belik, Stein, Asmundson, & Sareen, 2010). However, exposure to atrocities or having purposely injured or killed someone were associated with lifetime suicide attempts; peacekeeping and combat were not (Belik, Stein, Asmundson, & Sareen, 2009).

Sareen et al. (2008) found the population attributable fraction (PAF) for combat or exposure to atrocities and mental disorders to be 9% in men and 6% in women; few cases of mental disorders as a whole would have been prevented had deployment not occurred. Only PTSD had a sizable PAF for deployment (47% in men; 24% in women). Little of the burden of depression was attributable to combat or peacekeeping.

The primary strengths of these survey data are the high response rate (80%) and the precision with which mental disorders were assessed. The primary limitations are the limited assessment of specific deployment experiences and the inability to directly attribute individual cases of mental disorders to specific deployments.

The key finding of this study is that CAF personnel in 2002 had a substantial prevalence of a broad range of 12-month mental disorders, with depression (and not PTSD) being the most prevalent. Given that many of those who participated in the 2002 mental health survey would have ultimately gone on to deploy in support of the mission in Afghanistan, the primary implication of these findings to the present paper is that a substantial fraction who deployed likely had a history of mental disorder. The small contribution of past deployments to the overall burden of mental disorders suggested that the Afghanistan mission, too, might ultimately be associated with a lower than feared impact.

## Afghanistan era findings

### 2004 and 2008/2009 Health and Lifestyle Information Surveys

The Health and Lifestyle Information Survey (HLIS) is the CF's periodic health surveillance survey (Department of National Defence [DND], 2005; DND, 2010). The prevalence of 12-month depression in the RegF was 7.4% in 2008/2009 and no different (7.1%) from 2004. No significant changes in suicidal ideation or suicide attempts were seen from 2004 to 2008/2009—a period during which the threat level of the operation increased dramatically. In contrast, the prevalence of high-risk drinkers rose significantly from 13% in 2004 to 20% 2008/2009.

In 2008/2009, 8.1% of all respondents met screening criteria for PTSD (Prins et al., 2003); recent deployment was not a risk factor for PTSD. Having been diagnosed with PTSD or depression as a chronic health problem was reported by 3% for each condition. However, the prevalence of PTSD as a chronic, diagnosed condition rose from 2004 to 2008/2009 (from 0.4 to 3%); no change was seen for diagnosed mood disorders. The primary limitations of HLIS surveys are the modest response rate (~50%), and the usual survey-related limitations.

This survey was the first indication that despite the unusual demands of the mission, the prevalence of mental disorders in the CAF was stable, a finding that is consistent with other work showing a small overall contribution of deployment to MHPs in serving personnel (Sareen et al., 2008).

### The 2010 Veterans Affairs Canada Survey on Transition to Civilian Life

The Survey on Transition to Civilian Life (SCTL) surveyed personnel who released from the RegF from 1998–2007 (Thompson et al., 2011); this population thus brackets the pre-Afghanistan and Afghanistan eras. Eleven percent of the population reported diagnosed PTSD as a chronic condition; 10% reported other anxiety disorders, 20% reported depression or anxiety, and 3% reported other mood disorders (Thompson et al., 2011). The rate of anxiety disorder was higher than the adjusted rate for the Canadian general population (10% vs. 5%). Twelve-month suicidal ideation and suicide attempts were seen in 6 and 1%, respectively.

The primary strength of this study is its inclusion of the entire population of recently released personnel and its high response rate. The main limitation is use of self-reported diagnosed conditions as mental health outcomes; these are subject to substantial misclassification (Smith et al., 2008).

This study is important because it was the first complete look at mental health of modern Canadian veterans, many of whom would have participated in the mission in Afghanistan. While direct comparison with survey findings of serving CF personnel is impossible due to methodological differences, the high prevalence of self-reported MHPs as a chronic condition was the first firm indication that the burden of mental illness may differ substantially between serving personnel and veterans.

### The 2010 Operational Mental Health Assessment (OMHA-I)

The CF undertook an Operational Mental Health Assessment (OMHA-I) for Kandahar-based personnel on one troop rotation, in order to understand need for services while deployed (Garber, Zamorski, & Jetly, 2012). This survey was modeled after the US Army's Mental Health Advisory Team (MHAT) survey (Office of the Surgeon General, 2006), but the CF used less stringent cut-offs for disorders than those used in the MHATs. An important minority (8.5%) reported symptoms of traumatic stress, depression, or generalized anxiety. Traumatic stress symptoms were reported in 4.6%; depression was reported in 4.5%. Among sociodemographic variables, being unmarried was the only independent risk factor. Combat exposure and home-front stressors (e.g., failure of an intimate relationship) were independently associated with MHPs. Reservists were not at increased risk, neither were those who had had multiple deployments.

The primary limitations of the OMHA-I are its modest response rate (53%), the limited statistical power to detect small differences, the fact that it pertains to a single rotation in a specific operation, and the usual limitations of surveys.

For the present paper, the key implication of the OMHA findings is that it reinforced the role of combat exposure in MHPs of deployed personnel. The substantially lower prevalence of MHPs in deployed personnel relative to methodologically-comparable US findings (Office of the Surgeon General, 2006) points to the potential for mental health impacts of deployment to vary substantially from nation-to-nation.

### Enhanced Post-deployment Screening Data (2006–2010)

Data collected 3–6 months post-deployment during screening from 2006 to 2010 showed that an important minority report symptoms of MHPs (Zamorski, 2011a). Approximately 3.4% reported symptoms of PTSD; 3.5% reported symptoms of major depression at the time of their screening; 4.9% reported one or both of these problems. An additional 6.8% reported symptoms suggestive of other MHPs. High risk drinking was reported by 13% in the absence of a MHP and by 4% as an MHP comorbidity.

Being unmarried, of lower rank, and having high combat exposure were independently associated with PTSD and/or depression, while ResF status was not. Each additional deployment was associated with a 0.2 *percentage point* increase in the risk of PTSD and/or depression. From 2006 through 2009, the prevalence rate of PTSD and/or depression declined from 7.0 to 3.3% even though conditions on the ground did not change much. Half of those with symptoms of PTSD were already in care at the time of their screening, suggesting that mechanisms other than screening are facilitating early care.

The post-deployment screening data is relatively representative, given its high response rate (76%). However, responses are confidential, not anonymous, so the reported prevalence rates almost certainly represent a systematic underestimate of the true prevalence (Warner et al., 2011). In addition, there is no non-deployed comparison group to put these prevalence rates into perspective. Finally, it captures MHPs at only a single point in time in the first year after return from deployment, during which the prevalence of mental disorders appears to be increasing substantially (Thomas et al., 2010). Differences between these Canadian findings and those in the US (notably, the lack of a strong association of MHPs with ResF status or with multiple deployments) points to the need for caution in applying the results of studies in one military organization to another (Zamorski, Rusu, & Garber, 2014).

### 1995–2008 Regular Forces Suicide Surveillance

CF mortality surveillance data from 1995 to 2008 showed that the suicide rate for RegF males was 18–20 per 100,000 per year and was stable over time (Zamorski, 2010). The standardized mortality ratio (SMR) relative to the Canadian general population ranged from 0.72 to 0.83, reflecting a lower or similar rate of suicide in serving CF personnel relative to civilians. Ever having deployed was not a risk factor. The primary limitations of these data are small numbers of events, the inability to look at suicide rates in female personnel (due to small numbers of events), and the crude way in which deployment experiences were captured (ever vs. never deployed). These findings are significant in that they once again demonstrate divergence between US military findings of dramatically rising suicide rates over the past decade (Armed Forces Health Surveillance Center [AFHSC], 2012) and Canadian findings.

### 2011 Canadian Forces Cancer and Mortality Study

A retrospective cohort mortality study of approximately 190,000 individuals with RegF service from 1972 to 2006 identified 4,000 deaths over the follow-up period; injury and suicide were the two leading causes of death (Statistics Canada, 2011). Suicide mortality was similar to civilians in both men and women (SMR=1.01 and 0.99, respectively). However, among released personnel (veterans) there was a statistically significant increased risk of suicide in men (SMR=1.46) but not in women. Independent risk factors for suicide included male sex, age of less than 30 at the time of release, non-officers, less than 10 years of military service, service prior to 1987, and medical and involuntary releases. Deployment history has not yet been explored as a covariate, and analysis of in-service deaths has not yet been completed. As with analysis of MHP prevalence rates, these findings point to the potential between divergent mental health-related findings in serving personnel and veterans.

### 2011 Canadian Forces Base Gagetown Cohort Study

A retrospective cohort study of a single rotation of Afghanistan-deployed combat and combat support personnel at a single base (*N*=792) abstracted diagnoses and clinical attribution to military service from medical records (Sedge, Devlin, & Joshi, 2012). Twenty-three percent of the cohort was diagnosed with a service-related mental disorder (largely PTSD) over the 4 years after their return. Of the 20% of the cohort diagnosed with PTSD, 62% had temporary duty limitations, 15% had permanent duty limitations, and 8% had been medically released as a consequence of their service-related MHPs. Lower ranking personnel and those in the combat arms trade (and particularly combat engineers) had higher rates of PTSD.

The strength of this study is that it was the first to use clinical data (including a clinician's causal attribution) for previously deployed CF personnel. In addition, it is the only CF study on occupational outcomes of those with service-related PTSD. The lengthy follow-up period is another strength, though the large fraction of personnel with temporary duty restrictions means that the ultimate effect of PTSD on employability remains uncertain. The primary limitation of this study is that it pertains to a single rotation of high-risk personnel. The mismatch between the MHP prevalence rates in this study and those seen in previous studies (above) pointed to the need to look at mental health outcomes in the broader population of previously deployed personnel over a more prolonged follow-up period.

### 2011 Operational Stress Injury Cumulative Incidence Study

The studies described above represent a substantial body of scientific knowledge on the effect of recent deployments on mental health in the CF. However, there was a recognized need for a study that addressed their important limitations, specifically one that covered the full range of Afghanistan deployment experiences since the beginning of the mission in 2001; used a prolonged follow-up period; was based on the outcomes of the CF's detailed clinical assessments (as opposed to surrogate outcomes determined by questionnaires); used the entirety of the clinical information available to the assessing clinician to determine whether the diagnoses are related to the mission in Afghanistan; and analyzed time-dependent outcomes (such as diagnosis and outcome) using a time-to-event (survival) approach.

The Operational Stress Injury Cumulative Incidence Study reviewed the medical records of a random sample of 2,014 out of the 30,513 personnel who deployed in support of the Afghanistan mission for any period during 2001 through 2008 (Boulos & Zamorski, 2013). Thirty percent accessed specialty mental health services over a median follow-up period of 4 years. Eight percent of the population was diagnosed with Afghanistan-related PTSD, and an additional 5.5% were diagnosed with other Afghanistan-related mental disorders. As well, 1.2% were diagnosed with a service-related mental disorder not due to Afghanistan, and 4.5% had non-service-related disorders. Inspection of the survival curves showed the brisk presentation of cases in the first months and years after return from deployment; only after 6 or 7 years does the rate of new cases taper off substantially.

Cox proportionate hazard modeling identified these independent risk factors: high-threat deployment locations (Kandahar and to a lesser extent Kabul), low rank, and Army service. However, substantial rates were seen in those who deployed only to low-threat locations (6.5% for the Arabian Gulf and 3.3% for the United Arab Emirates). Multiple deployments, female sex, and Reserve status were not independent risk factors, though power to detect small differences was limited.

The strengths of this study are listed above. Its principal limitation is that it could only capture diagnoses made by the CF over the follow-up period. While the rigor and standardization of the CF's diagnostic process militate against misdiagnosis, the reliability of deployment-specific attributions is unknown.

The primary implications of this study are first, that a large fraction of personnel will seek mental health care in the aftermath of a difficult deployment, providing a substantial opportunity to diagnose and treat deployment-related MHPs. Second, cross-sectional symptom surveys (such as post-deployment screening questionnaires) substantially underestimate the burden of deployment-related illnesses. Finally, this study drives home that while combat exposure is a powerful driver of deployment-related MHPs, a meaningful prevalence of MHPs will be seen even in groups with little or no combat exposure.

### TBI studies

At the time of their post-deployment screening, 5% of personnel deployed in support of the mission from 2009 to 2010 reported an injury with alteration in mental status (TBI) while deployed (Zamorski, 2009). Most TBIs fell into the mildest form of the TBI spectrum (i.e., were associated with at most brief loss of consciousness). Combat exposure was a strong risk factor for TBI.

Of those with TBI, approximately one-quarter had multiple persistent “post-concussive” symptoms. Multiple post-concussive symptoms had little independent association with TBI. Instead, persistent symptoms were intensely associated with MHPs. As a result, most of those with multiple “post-concussive” symptoms were in the group *without* TBI.

The primary limitation is that the screening test used has never been completely validated against an objective clinical standard (Schwab et al., 2007). In addition, these findings apply only to deployed personnel, making it impossible to place deployment-related TBI in the context of the overall population burden of TBI (most of which are unrelated to deployment (AFHSC, 2007)). Finally, the usual survey-related biases apply.

## Synthesis

The first and foremost message of these studies is that most personnel returning from a mission (even a very difficult one) are well, but an important minority will suffer from MHPs after their return. Many of these will be clinically attributed to the mission. PTSD and its co-morbidities predominate among deployment-related mental disorders in clinical populations, but a number of other non-trauma-related disorders (e.g., social phobia) are associated with traumatic deployment experiences in surveys. Typical risk groups for mental disorders in military are seen in CF personnel. However, CF ResF service is not a risk factor for MHPs during or after deployment. The primary driver of deployment-related MHPs is trauma exposure. Nevertheless, meaningful rates of deployment-related MHPs are seen in the large numbers of personnel deployed to low-threat areas, so the population burden will still be substantial.

Understanding the health effects of a deployment is impossible without detailed knowledge of deployment experiences. For those with multiple deployments, the effects of the most recent one need to be understood in the context of previous ones. In the CF, little or no association between number of deployments and MHPs is seen, suggesting that the CF's policies, programs, and services that are designed to mitigate cumulative effects are working. This may, however, reflect the heterogeneous nature of deployments. Using “total number of career deployments” or “ever vs. never deployed” as indicators of psychological toxicity is a pragmatic but crude approach; even using “ever deployed on a specific operation” has limitations when the extent of trauma exposure varies so much from person to person.

A substantial fraction of personnel will seek mental health care after return from deployment. However, many present for care only years after their return, pointing out the need for further intervention to overcome barriers and shorten the period of time between disorder onset and first care. Until those aspirations are realized, military and veteran organizations need a robust mental health capacity for many years after the deployment is complete.

For the CF population as a whole, deployment contributes relatively little to overall population burden of mental disorders. Superficially, the finding of a low PAF for deployment and mental disorders conflicts with the finding that most mental disorders diagnosed in Afghanistan-deployed personnel were attributed to the mission by their clinician. However, these are very different ways of making attributions: The PAF approach explicitly takes into account the multiplicity of factors that contribute to MHPs. The clinical approach would attribute PTSD characterized by intrusive re-experiencing of deployment-related events as a deployment-related mental disorder, even if the client has other strong risk factors for MHPs.

Suicide is clearly an important public health problem in military organizations, making suicide prevention a public health priority (Zamorski, 2011b). While difficult operations can have a serious long-term effect on mental health, in the CF this does not translate into a demonstrably higher risk of suicide for the CF population as a whole, at least not while in service. Deployment-related MHPs can, of course, contribute to suicidal behavior in individual cases. After release, there does appear to be an increase in suicide risk compared to civilian controls. This points to the potential for differential effects in serving vs. veteran populations and to the need for effective suicide prevention in the veteran population.

A minority of personnel deployed on a combat operation have a meaningful risk of sustaining a TBI. The least severe forms of TBI will predominate. Persistent “post-concussive” symptoms appear to have little or no specificity for mild TBI. Instead, they appear to be tightly related to mental disorders. This argues for careful assessment of mental disorders in those with physical symptoms, whether or not they have sustained mild TBI (Garber, 2008).

## Conclusion

The data presented above paint a largely encouraging picture of mental health in the CF: Most personnel are well, despite the demands of military service. The incremental toxicity of repeated deployments is kept to operationally acceptable levels. The prevalence of post-deployment MHPs appears to be declining, not increasing, and suicide rates are stable. Many of those who do develop a mental disorder do seek care. TBI occurs more often than we would like, but most are not troubled by ongoing symptoms.

Getting a complete picture of the impact of a military operation requires a number of different studies with different methods. The studies presented above provide different numbers (a source of consternation to our leaders and the media), but taken as a whole, they do cohere: More stringent criteria lead to lower prevalence rates, point prevalence rates are lower than period prevalence rates, trauma-exposed populations have higher risk, consistent risk factors are seen, and so on. While our findings largely pertain to the conflict in Afghanistan, our participation there was diverse enough that the mental impact on subgroups has much to say about the likely impact of past and future conflicts.

Remaining knowledge gaps on the impact of deployments on MHPs in the CF are being closed by additional studies. For example, data on resilience collected from recruits is being linked to a broad range of later health outcomes (Lee, Sudom, & Zamorski, 2013). Data collection for another population-based prevalence survey (similar to the one done in 2002) was completed in 2013. This survey will assess the divergent impacts on need for care of the mission in Afghanistan and of the renewal of our mental health system. It will help also situate deployment-related mental health impacts in the broader context of determinants of mental health in the CF. A longitudinal component to the 2002 mental health survey (Statistics Canada, 2004) is being developed to better understand transitions in mental health and their correlates over more than a decade. Studies looking at clinical and organizational outcomes of personnel with service-related MHPs and with TBI are underway. Veterans Affairs Canada has also recently completed data collection on its “Life after Service Survey,” another health survey of modern veterans.

Assessing the psychological impact of deployments has intrinsic value: Those who ensure our security at great risk deserve to have the full measure of their sacrifice documented. Epidemiological studies also help understand the etiology of mental disorders, identify risk groups, and assess the effectiveness of prevention and control measures. As such, they are an essential activity for military organizations.

However, the CF is fast approaching a point where we will have, for the purposes of prevention and control, a sufficiently complete picture of the mental health impact of the mission in Afghanistan. As such, the time is coming to focus on other research objectives, such as understanding the performance of our mental health care system, the effectiveness of our mental health education system, and the impact of our mental health screening programs. Development and evaluation of new prevention or treatment approaches is urgently needed given that too many patients fail to tolerate or benefit fully from the best available therapies (Mendes, Mello, Ventura, Passarela, & Mari, 2008; Stein, Ipser, & McAnda, 2009). The growing economic burden of mental health services calls for the development of more efficient treatment approaches. In short, our research focus needs to change from *counting* the mentally ill and injured to *helping them recover*.
